# The IPD Project: a centralised resource for the study of polymorphism in genes of the immune system

**DOI:** 10.1007/s00251-019-01133-w

**Published:** 2019-10-22

**Authors:** Giuseppe Maccari, James Robinson, John A. Hammond, Steven G. E. Marsh

**Affiliations:** 1 The Pirbright Institute, Pirbright, Woking, Surrey, UK; 2 Anthony Nolan Research Institute, Royal Free Hospital, London, UK; 3 Present Address: UCL Cancer Institute, Royal Free Campus, London, UK

**Keywords:** HLA, MHC, IPD, Database, Nomenclature

## Abstract

The Immuno Polymorphism Database (IPD), https://www.ebi.ac.uk/ipd/, is a set of specialist databases that enable the study of polymorphic genes which function as part of the vertebrate immune system. The major focus is on the hyperpolymorphic major histocompatibility complex (MHC) genes and the killer-cell immunoglobulin-like receptor (KIR) genes, by providing the official repository and primary source of sequence data. Databases are centred around humans as well as animals important for food security, for companionship and as disease models. The IPD project works with specialist groups or nomenclature committees who provide and manually curate individual sections before they are submitted for online publication. To reflect the recent advance of allele sequencing technologies and the increasing demands of novel tools for the analysis of genomic variation, the IPD project is undergoing a progressive redesign and reorganisation. In this review, recent updates and future developments are discussed, with a focus on the core concepts to better future-proof the project.

## Introduction

Genetic variation often underpins differential disease susceptibility between individuals and populations. Several genes that help orchestrate the response to pathogens with core functions at the heart of the immune system evolve extremely rapidly under intense selection pressure. For example, the major histocompatibility complex (MHC) is the most variable region between mammalian genomes, containing genes with extremely high levels of polymorphism present in various configurations on different haplotypes. This level of diversity (MHC and other immune-related loci) requires an unambiguous nomenclature system and highly curated sequence data to systematically study and interpret the functional consequences of this variation (Klein et al. 1990; Ellis et al. 2006). To this end, the Immuno Polymorphism Database (IPD) project was established in 2003 by the HLA Informatics Group of the Anthony Nolan Research Institute (Robinson et al. 2003, 2005) to provide a centralised repository of expertly curated and annotated sequences.

Following the success of the IMGT/HLA Database in providing a unique resource for the study of human MHC, the IPD project was built as a set of independent, manually curated and highly informative databases. The key aims are to extend the study of polymorphic genes that function within the immune system and to facilitate the analysis and comparison of nucleotide and protein sequences by accommodating all the information under the same structure.

Submitted sequences are manually curated by a panel of experts in the field, overseen by specialised nomenclature committees, generating accurate data and a high level of annotation. The unified nature of the IPD project allows the diffusion of data in a standardised format, facilitating the comparison of sequences between different species and availability of bioinformatics tools. The IPD project is distributed in collaboration with the European Bioinformatics Institute (EBI) facilitating integration with the array of tools and data provided and hosted by the EBI. The available databases in the IPD project are summarised in Table 1.

**Table 1:** Databases composing the IPD project

Name	Description	First released	Reference
IPD-IMGT/HLA	Human major histocompatibility complex and related genes	1998	(Robinson et al. 2015)
IPD-KIR	Human killer-cell immunoglobulin-like receptors	2003	(Robinson et al. 2010)
IPD-MHC	Non-human major histocompatibility complex	2002	(Maccari et al. 2017)
IPD-NHKIR	Non-human killer-cell immunoglobulin-like receptors	2018	(Robinson et al. 2018)
IPD-HPA	Human platelet antigens	2003	(Metcalfe et al. 2003)
IPD-ESTDAB	The European Searchable Tumour line Database (ESTDAB) and cell bank	2003	(Robinson et al. 2009)

**Fig. 1:**
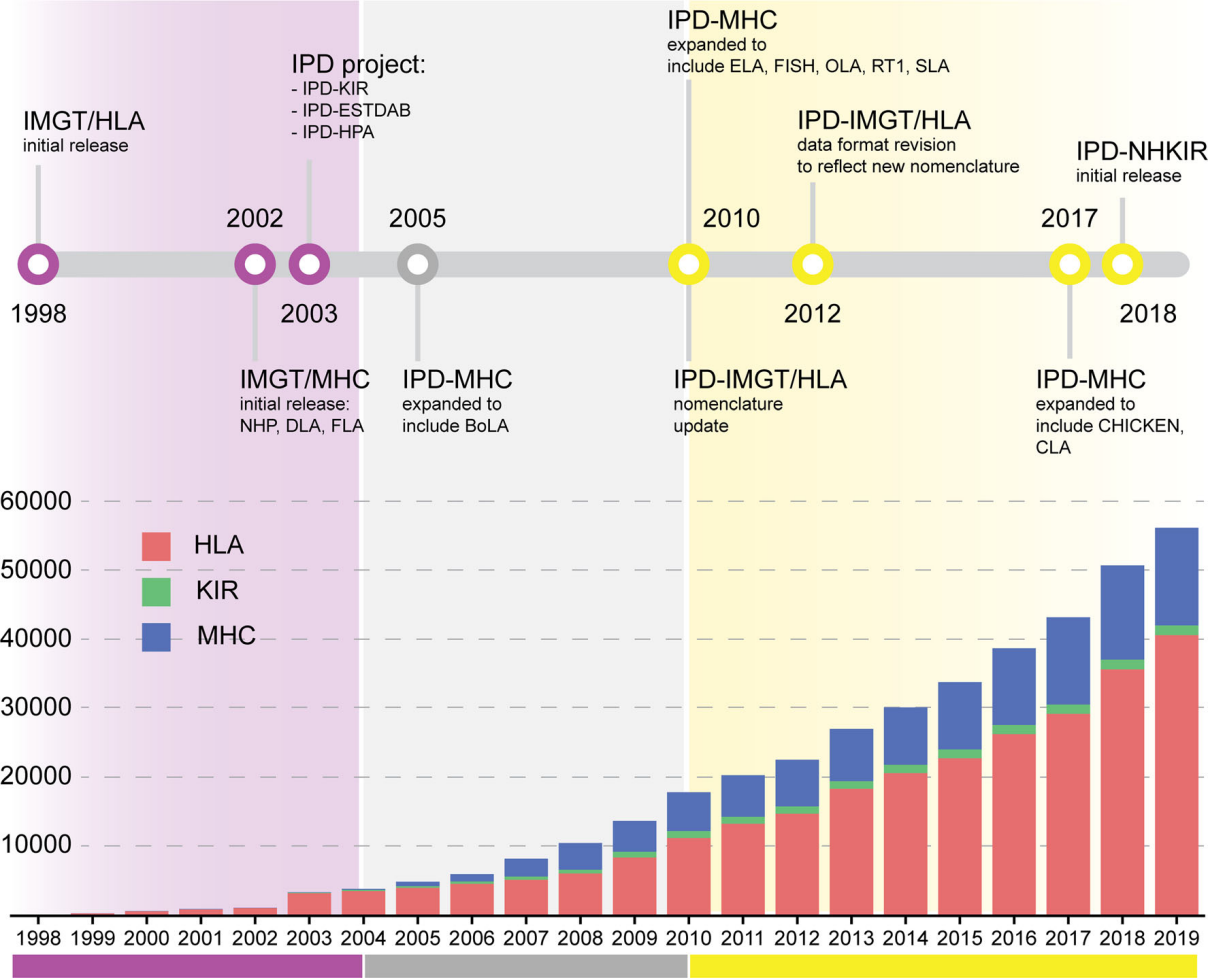
IPD project timeline. The IPD project has grown in number of sequences and project hosted in order to reflect the community requirements and the improvement in allele typing technologies (top panel). The IPD-IMGT/HLA Database was first released during the last period of the serological typing era (not shown), with the introduction of the other components of the IPD project during the second era where DNA-based methods for typing were first being developed (purple). High-resolution probe-based typing (grey) brought a substantial increase of the number of submitted sequences, escalating during the current period of allele typing, dominated by high-throughput sequencing-based typing techniques (yellow).

Recent technical advances in high-throughput sequencing have driven exponential increases in data volume and quality. Alongside continual advances in bioinformatics and statistical tools, new approaches to studying the immune gene repertoire in any species have continued to develop, providing an unprecedentedly high-resolution picture of the immune repertoire. Consequently, the IPD project has faced the challenge of constant growth in size, both from sequence numbers and taxonomic groups, and increased demand for data access and unified bioinformatics tools (Fig. 1). By looking at the influx of submitted data since IPD-IMGT/HLA was released, it is possible to identify four major periods in the IPD project history that have been driven by advances in allele typing techniques.

Serological typing dominated the initial period before the first database release (data not shown), when DNA sequencing techniques were still in development. DNA-based methods for allele typing dominated the beginning of the twenty-first century, with most of the techniques based on sets of oligonucleotide probes, with a limited potential to detect novel alleles. Subsequently, high-resolution probe-based typing was developed and first applied to large panels of samples, greatly improving the potential to identify novel alleles. Since 2010, sequence discovery has been dominated by the use of high-throughput sequencing techniques able to detect any novel allele to base pair resolution (Robinson et al. 2017).

The increasing number of novel alleles being submitted and the extension of allele names due to the incorporation of genomic regions generates an unsustainable amount of work for the curators, often relying on local analysis. The lack of a centralised organisation hampered the development of unified tools for data submission, curation and comparison, making the development of more advanced bioinformatics tools impossible. The IPD project had to be updated to react to this demand to remain a useful tool for research and also maintain the unified standards of the official data repository.

Since 2016, a progressive redesign and reorganisation of the IPD project has been undertaken, including the release of new analysis tools, the improvement of the curation pipeline and the addition of the IPD-NHKIR Database to the IPD family. In this review, recent updates of the IPD project are listed and future developments are discussed. In particular, an overview of the required changes in the IPD organisation and core concepts to future-proof the project is presented. The overall aim is to provide a universal bioinformatics framework that is flexible enough to deal with the immune system gene polymorphisms in human and all vertebrates.

## IPD-IMGT/HLA

The IPD-IMGT/HLA Database is a specialist repository for the allelic sequences of the genes in the HLA system, the human major histocompatibility complex. This complex of ∼ 4 megabases is located within the 6p21.3 region of the short arm of human chromosome 6 and contains over 220 genes involved in antigen presentation to T cells. It is one of the most complex and polymorphic regions of the human genome (Robinson et al. 2017). The HLA comprises several highly polymorphic genes that play an important role protecting the organism against invading pathogens and is fundamental to the outcome of cell and organ transplants (Petersdorf 2004; Trowsdale and Knight 2013).

HLA allele sequences can differ from each other by as little as a single-nucleotide polymorphism (SNP), and even small differences between the alleles of prospective transplant donors and recipients can make the difference between a successful transplant, graft failure and death. As a result, given the large impact of sequence variation on the outcome of a transplant, it is vital to maintain high standards of both control and curation. For this reason, the naming of new allelic sequences and their quality control is the responsibility of the World Health Organization (WHO) Nomenclature Committee for Factors of the HLA System (Marsh et al. 2010), with the IPD-IMGT/HLA Database acting as the official repository and primary source of up-to-date and accurate HLA sequences.

The IPD-IMGT/HLA Database was first released in 1998 as part of the IMGT project on the EBI server. Over the last 20 years, the project has faced many changes and expansions, including the addition of tools for the analysis and manipulation of HLA sequences. The rapid uptake of high-throughput sequencing techniques since 2010 triggered a dramatic increase in the number of sequences deposited into the database, providing not only novel sequences but also extending and filling gaps within existing entries. In addition to sequence data, a large amount of background metadata is held on the source material from which the sequences were derived. This further supports the matching of donors and recipients of transplants. While the impact of this new data in a clinical setting is still being determined (Mayor et al. 2019), the bioinformatics challenges of curating and managing this new efflux of data is currently being addressed.

## IPD-MHC

Unlike the IPD-IMGT/HLA, the IPD-MHC Database contains a number of different species that each require specific expertise for curation. Consequently, this database is the result of several species-specific nomenclature committees and individual curators overseen by the Comparative MHC Nomenclature Committee. This standing committee is supported by the International Society of Animal Genetics (ISAG) and the Veterinary Immunology Committee (VIC) of the International Union of Immunological Societies (IUIS) (Ellis et al. 2006; Ballingall et al. 2018). Since its first release in 2003, the database has grown in number of hosted taxonomic groups concerning food security, animal companionship or medical research; cattle (BoLA) (Hammond et al. 2012), teleost fish (FISH) (Yamaguchi and Dijkstra 2019), rat (RT1)(Günther and Walter 2001), sheep (OLA) (Ballingall et al. 2011), swine (SLA) (Ho et al. 2009), horse (ELA) (Tseng et al. 2010), non-human primates (NHP) (de Groot et al. 2012) and dog (DLA) (Kennedy et al. 2007).

In 2015, the IPD-MHC project was supported by a UKRI-BBSRC Bioinformatics and Biological Resource grant with the aim of updating and expanding the database to include even more taxonomic groups of economic and scientific interest. As a result, the project was reorganised in order to host all the taxonomic groups under a unified database, and effort was made to future-proof the project with an eye to new technologies. The recent changes allow the collection and comparison of genomic and non-genomic sequences and provide tools for the inter- and intraspecies comparison of allele variation, facilitating both small and large MHC groups. This enhanced functionality has required a new level of standardisation in the MHC nomenclature between species and groups to allow an unambiguous inter- and intraspecies comparison of alleles and encouraged the MHC Nomenclature Committee to draft an improved set of guidelines (Maccari et al. 2018) covering MHC variation at genomic level. Furthermore, the reorganisation of the IPD-MHC Database spurred the realisation of new analysis tools and the revision of existing ones. A novel algorithm for the inter- and intralocus alignment was introduced, allowing for the first time the comparison of loci from different species in real time and the download of the aligned sequences for further studies and analysis. A sequence matching tool provides the user with the ability to easily compare non-published sequences with the curated dataset in the IPD-MHC Database, generating a report of the most similar sequences across the whole spectrum of species in the database.

Due to the improvement in sequence length and quality given by high-throughput sequencing, haplotype data is now available for an increasing number of organisms, allowing a deeper understanding of the complexity and recombination. Haplotype data provides an essential resource to precisely define disease-associated polymorphisms within the MHC and can be used as reference for the assembly of high-throughput sequencing data. To this end, the IPD-MHC Database will introduce manually curated haplotype data for each taxonomic group, where an overview table will show haplotype data as well as haplotype frequency and allele names.

**Fig. 2:**
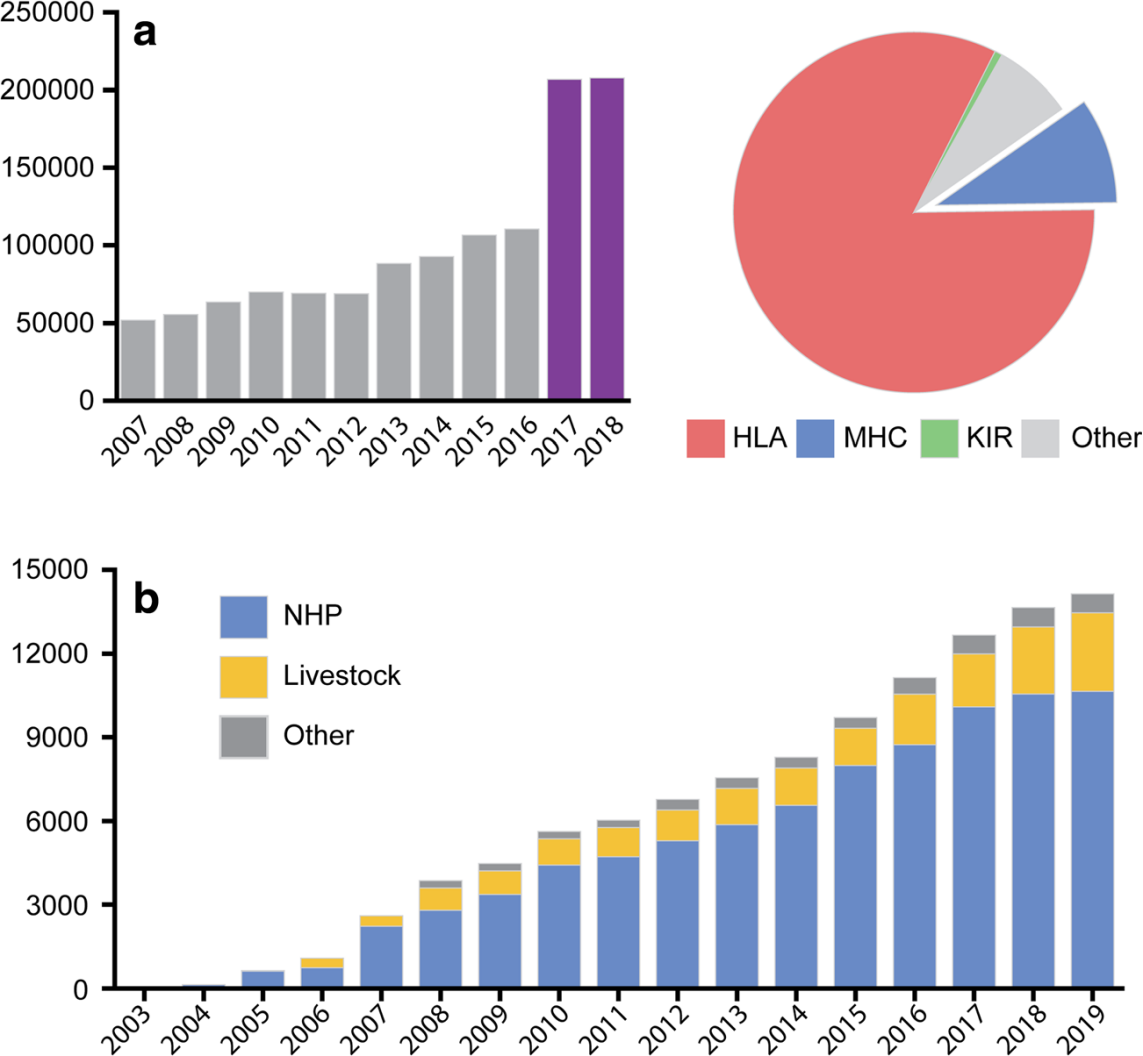
Distribution of IPD-MHC data. **a** Number or visits by year; left panel: IPD-MHC number of visits (grey, before update; violet, after update); right panel: number of visits in 2019 of the IPD project. **b** Distribution of submissions over the years.

Following the advance in data organisation and analysis tools, the IPD-MHC Database is generating a renewed interest, perceivable both in the database traffic and in the increasing amount of submitted data. Since its update in 2016, the number of visits per year doubled (Fig. 2a), accounting for nearly 10% of the overall traffic generated by the IPD project. Figure 2b shows the number of submitted sequences highlighting the increase, especially for livestock and farmed species. Farmed species are an integral component of the food security agenda and improving their genetics has enormous potential to increase sustainable production and reduce economic burdens. For this reason, two novel taxonomic groups were recently introduced, providing an official nomenclature for MHC sequences of chicken (CHICKEN) (Maccari et al. 2017) and goat (CLA) (Ballingall and Todd 2018). This has ensured that IPD provides reference data for the vast majority of farmed species in the IPD-MHC Database.

## IPD-KIR

The model set by the IPD-IMGT/HLA Database has also been applied beyond the MHC—such as the IPD-KIR Database. Killer-cell immunoglobulin-like receptor (KIR) genes are members of the immunoglobulin super family (IgSF), previously known as the killer-cell inhibitory receptors, they are highly polymorphic at both allelic and haplotypic levels (Garcia et al. 2003). KIRs are composed of two or three Ig domains: a transmembrane region and cytoplasmic tail, which may be short (activatory) or long (inhibitory).

Given the complexity in KIR regions and sequences, the KIR Nomenclature Committee was established in 2002, in order to undertake the naming of human KIR genes and their allele sequences. The first KIR Nomenclature report was published in 2003 (Marsh et al. 2003), coinciding with the first release of the IPD-KIR Database. The initial release included just 89 officially named human KIR alleles, as of August 2019, there are now almost 1,000 alleles, coding for over 500 unique KIR protein sequences.

Multiple studies have demonstrated an increase in the transplant outcome in patients with donors presenting a favourable KIR type (Ruggeri et al. 1999; Cooley et al. 2010), highlighting the importance of KIR matching in transplantation outcome as an additional selection criteria.

With the improvement of cost-efficient high-throughput sequencing techniques, the volume of available data is increasing, providing a volume of data never available before (Wagner et al. 2018). For this reason, as new influxes of high-throughput data are generated, the organisation of a centralised resource for the curation of KIR sequences is becoming even more imperative.

## IPD-NHKIR

The KIR region has been studied in a number of non-human primates and is characterised by high levels of allelic polymorphism and haplotypic polymorphism in the number of genes and extensive duplication and recombination (Hammond et al. 2016). These features have made it difficult to assign orthologues and have led to a number of different nomenclature systems being used to name genes and alleles. The increase in number of sequenced KIR alleles generated a growing interest for a common repository of non-human KIR sequences. As a result, the IPD-NHKIR Database was released in 2018, together with a set of guidelines for the sequencing and submission of non-human KIR alleles. The IPD-NHKIR nomenclature and guideline are based on the human KIR database and incorporate species-specific modifications to accommodate interspecies variation. The first release of the database includes alleles from rhesus macaque (*Macaca mulatta*), chimpanzee (*Pan troglodytes*), orangutan (*Pongo abelii* and *Pongo pygmaeus*) and cattle (*Bos taurus*), for a total of 266 and 23 NHP and BoLA alleles, respectively (Sanderson et al. 2014; Robinson et al. 2018). Submissions to the IPD-NHKIR Database are handled by the recently introduced IPD submission tool, allowing users to contribute to the expansion of the database. This provides an example of how the modular organisation of the IPD project is beneficial for its sustainability, allowing the reutilisation of existing parts to expand and implement new sections.

## IPD-ESTDAB and IPD-HPA

The remaining two projects are databases representing a cell-bank of human cells (ESTDAB) and a SNP catalogue for Human Platelet Antigens (HPA). The two databases have been part of the IPD project since 2003 and represent legacy systems that are no longer under active development but are provided to the community for reference purposes.

## Discussion and future development

Modern genetic data analysis is often organised to follow a set of chronological tasks: data is acquired in the form of genomic sequence and cataloguing genetic sequence variation; this variation is then used to examine large populations, then variation is correlated with a specific phenotype. In the context of the IPD project, variation is correlated generally speaking with disease susceptibility or resistance, and in the specific case of IPD-IMGT/HLA to transplant compatibility. While the technology to acquire vast amounts of genetic data is now well established and continues to expand, the analysis of such data is still challenging, especially for highly polymorphic genomic regions. The key aim of the IPD project is to aid the analysis and interpretation of the immune repertoire, by providing high-quality manually curated data for the analysis and comparison of genomic variation in one of the most polymorphic regions. By accommodating related systems in a single database, data can be made available in common formats aiding use and interpretation.

To enforce the data centralisation concept, a centralised submission tool has been released, with the aim to provide the IPD project with a single tool able to handle the various requirements of each database. The data collected and curated by the IPD project can be generalised in a number of common fields, where the DDBJ/ENA/GenBank accession number is the primary requirement and allows to connect the input sequence to a single organism and locus. This is of particular importance for the non-human databases, where wrong taxonomic information can cause ambiguity in the allele nomenclature. Other fields are automatically extrapolated from the provided accession number, including the sequence annotations and the experimental methodology. These fields are editable by the user and are automatically validated during the submission. Additional database-specific fields may be collected either to provide the database curators with specific information required for data validation or to show additional information. For example, the cattle section of the IPD-MHC Database requires a non-mandatory ‘breed’ field, while the non-human primates section presents a ‘colony’ field. This centralised IPD submission tool is currently adopted by the IPD-MHC and IPD-NHK and will be extend to the other components of the IPD project.

The recent redesign of the IPD project allows a more consistent and accurate analysis of data, providing high-quality data and facilitating the comparison of the immune variation. Synergistically, this in turn provides the different nomenclature committees with a standardised tool for the analysis and naming of alleles. Furthermore, the availability of high-quality, manually curated data will spur the development of tools for the analysis and interpretation of allele variation, expanding the existing toolset.
